# A comprehensive overview of breeding strategy to improve phenotypic and quality traits in *Valeriana jatamansi* Jones

**DOI:** 10.1016/j.heliyon.2023.e18294

**Published:** 2023-07-22

**Authors:** Rahul Dev Gautam, Ajay Kumar, Satbeer Singh, Ramesh Chauhan, Ashok Kumar, Sanatsujat Singh

**Affiliations:** a Academy of Scientific and Innovative Research, (AcSIR), Ghaziabad, 201002, India; b Agrotechnology Division, Council of Scientific and Industrial Research-Institute of Himalayan Bioresource Technology, Palampur, Kangra (H.P), India

**Keywords:** Breeding, Essential oil, Genetic improvement, Quality, Valeriana, Variations

## Abstract

*Valeriana jatamansi* is a high value perennial herb that grows at an altitude of 1000–3000 MASL in the Indian Himalayan Region and is used in the Ayurvedic, Unani and Chinese systems of medicine. The plant extracts and essential oil (EO) obtained from its roots are used in the pharmaceutical, aromatic and flavouring industries. On account of high global annual demand and lack of organized cultivation of this herb, it is mostly collected from the wild causing depletion of the natural populations and also leading to variable produce making it unsuitable for industrial use. Therefore, to promote its cultivation, it becomes imperative to develop varieties with uniformity. Significant variations have been reported in ***V. jatamansi*** populations in terms of underground biomass accumulation, essential oil and valepotriates indicating potential of genotypic variability for effective selection. The review focuses on the diversity and variations at inter- and intra-population levels for phenotypic traits, variations for different active constituents and scope of improvement through selective breeding in *V. jatamansi*. The species has cross-pollinated breeding behaviour on account of floral dimorphism and presents unique opportunities for development of homozygous progeny lines through controlled self/sib-pollination by applying the breeding methods described in the review for population improvement. The germplasm resources of unique and improved selections can be maintained clonally to ensure their true-to-type identity. This review article was framed in the year 2022 after thoroughly studying the literature from the year 1919–2022. The study focuses on the variations in ***V. jatamansi*** which could be used to maximize the production through various breeding techniques for biomass and yield of different active constituents to meet the requirements of pharmaceutical and aroma industries.

## Abbreviations

MASL: Meters Above Sea Level
RAPD: Random Amplified Polymorphic DNA
ISSR: Inter Simple Sequence Repeats
EO: Essential oil
Mbp: Megabase pair
MT: Metric Ton
gm: gram
cm: centi meter
NAA: 1-Naphthaleneacetic acid
2,4-D: 2,4-dichlorophenoxyacetic acid
IBA: Indol Butayric Acid
MS: Murashige and Skoog medium
BAP: 6-Benzylaminopurine

## 1 Introduction

*Valeriana jatamansi* Jones (synonymous with *Valeriana wallichii* DC.) commonly known as Indian valerian or Tagar belongs to the family Caprifoliaceae (Earlier Valerianaceae; [1]). It is a perennial aromatic medicinal herb native to the Himalayas which is utilized for medicinal purposes in China and India and also as a spice in India [2]. It is found at an altitude of 1,000 to 3,000 MASL [3] mostly on moist and shady slopes. An Indian physician first quoted the word ‘Valerian’ in the 9th century from the word ‘Velo’ which means ‘powerful drug’ [4]. While in Latin, ‘Valerian’ is derived from the word ‘Valere’ which means to have clinical and aromatic properties [5]. It is used for various medicinal purposes due to the presence of valerenic acid and valepotriates isolated from rhizomes and roots which are psychopharmacological agents and used for drug preparation [5]. The plant holds potential for a range of activities like anticancer, antifungal, antibacterial, anti‐inflammatory, anticoagulant, antioxidative, hepatoprotective, antiprotozoal and neuroprotective properties due to the natural occurrence of valepotriates as active ingredients [6].

*Valeriana jatamansi* draws a long history among the ancient and modern medicinal systems. Various ayurvedic formulations are prepared using the extracts of the species [7] like ‘Divya Kesh Taila’ by Patanjali, ‘Sleep Health’ by Foresta Organics and ‘Sleep Sure’ by Cureveda. Epilepsy, hysteria, and urinary troubles can be treated through the use of this species [8]. Roots and rhizomes are used for hair blackening, perfumes, incense and to remove the foul smell of mouth [9, 10]. The roots are used for treating troubles related to blood, liver, head, eye, kidney ulcers, spleen, wounds, dry cough, intermittent fever, asthma, chronic fever, and cardiac debility [11]. In case of extreme headache, leaves can be crushed and used on the forehead [10]. The plant is used for curing skin diseases, sciatica, emmenagogue anxiety obesity, failing reflexes, insanity, hysteria, neurosis, snake poisoning and as a tranquillizer [12]. *V. jatamansi* is reported to improve Alzheimer's disease, learning, memory and Parkinson's disease [13]. Effect of total iridoids of *V. jatamansi* on the brain-gut axis in a mouse model in form of an anti-depressant has been reported by [14], while animal and clinical studies suggested its potential in ameliorating central nervous system depression [15]. Recently [16], the effectiveness of EO against the cigarette beetle, an insect of stored products. EO and extract from species are used especially for flavouring tobacco, honey, and beer in the pharmaceutical, flavour, and fragrance industries [17]. Traditionally, it is used in conditions such as scorpion stings and jaundice [18]. The acceptance of alternative and complementary medicine is the reason for the increasing demand for species worldwide. Among the 178 traded medicinal plants *V. jatamansi* has been traded in high volumes of over 100 MT/year [9, 19].

The type of habitat and altitudinal range mainly affects the genetic diversity of the crop [20]. More genetic heterozygosity was observed in *V. jatamansi* than in *V. ciliata* [21]. A large extent of information is available for variations among the natural populations and on analysing the different populations for phytochemicals, chemical diversity was also noticed among the different populations [22, 23, 24]. Major research work on the characterization of *V. jatamansi* phytochemicals is being carried out [[25], [26], [27], [28], [29], [30]] but progress on breeding aspects is still lagging. *Valeriana jatamansi* exhibits unique floral dimorphism which needs to be studied in detail to utilize it as a breeding tool in hybridization programs. Therefore, the present review analyses the diversity and variations among and within the natural populations of *V. jatamansi* for phenotypic traits, phytochemicals, the role of floral biology in pollination and breeding strategy for selective improvement.

The review attempts to collect the scattered information on various breeding tools and associated chemical parameters which are lined up in this review article. The review aims to focus on breeding strategies that could be applied for the varietal development program in ***V. jatamansi***. The improved varieties can further be used beneficially for the aromatic and pharmaceutical industry.

## 2 Material and methods

The literature on *V. jatamansi* and other related crops was collected online from various sources like Google Scholar, PubMed, Science.gov and Semantic Scholar. The literature from year 1919–2022 was collected and thoroughly studied during year 2022 to frame useful information from it. The information was then used for writing review article. The presented review contains 129 references, 4 tables and 6 figures.

## 3 Diversity and variations

The genus *Valeriana* has over 350 species worldwide [20] of which 12 species are endemic to India [4] and *V. jatamansi* is believed to have evolved recently [31]. Several evolutionary factors that include reproduction mode, mating system, natural selection, gene flow and seed dispersal affect the genetic diversity of the species [32]. The genomic size of *V. jatamansi* is reported to be 3.01 Mbp [33]. Various markers like RAPD and ISSR have been used by different researchers for the genetic diversity analysis of *V. jatamansi.* Polymorphism can be detected by using SSR markers and many alleles at a single locus can be identified by using microsatellites [34].

Diversity is controlled by genetic conditions which directly affect the EO and valepotriates; moreover, maximum genetic diversity is found to be at lower altitudes and keeps on decreasing as the altitude increases [35, 36]. Kumar et al. [37], evaluated the genetic diversity of the plants from the northern Himalayas and reported moderate to high genetic variability among different genotypes using RAPD markers. Jugran et al. [38], investigated the antioxidant activity with the use of ISSR markers and identified the population with the highest antioxidant properties which indicated that the biochemical traits are influenced at the genetic level.

More variations were reported within a population and very less (7%) among populations whereas gene flow was observed in some of the populations studied [31]. Populations from the Western Himalayas have also been studied for average gene diversity per locus and Nei's genetic diversity index has been reported to be 0.25–0.37 [36]. Singh **et al.** [39] studied the polymorphism using microsatellite markers and most polymorphic primers were mononucleotide repeats followed by di-, tri-, hexa- and tetra nucleotides. Nei's genetic diversity index was reported to be 0.28–0.48. A high level of genetic diversity will provide a wide genetic base and contribute to the fitness of the populations that could be used for breeding varieties with wider adaptability.

### 3.1 Botanical description

*Valeriana jatamansi* is a perennial herb and its height varies in the field and natural conditions. The plant height in the western Himalayas varied among the female and hermaphrodite plants. Among the female plants, plant height was found to be 48.4–80 cm while 44.2–68.2 cm in the case of hermaphrodite flowers [40]. However, Mukherjee and Chakraborty [41] reported that female plants are shorter in height than hermaphrodite plants. Plant height varies according to environmental conditions [40]. Whereas, in the Eastern Himalayas different height ranges of female and hermaphrodite flowers were reported by Chakraborty *et al.* [42] where female plants were in the range of 8–18 cm and hermaphrodite plants were in the range of 9–15 cm. The inflorescence is a corymbose type having a small cluster at the pinnacle with delicate green-coloured leaves at the apex of the petiole. Chakraborty *et al.* [42] selected nine lines based on morphological characterization which was carried out on a type of leaf margin and time of flowering. Phenotypically, the populations were characterized based on plant height, the number of leaves/plant, rhizome length, rhizome width and seed yield/plant for each sex type.

Two types of leaves are found in *V. jatamansi*, base and cauline. Base leaves are found during the vegetative phase while cauline leaves are formed during the reproductive phase with the initiation of flowering buds when the flowering stalk develops. Pinnate venation is found in leaves with cordate or ovate shapes. Variable leaf margins are found in the leaves which may be entire, sinuate, serrate, dentate or wavy having acute leaf apex [43]. There is the presence of paracytic stomata on both abaxial and adaxial surfaces of the leaf, however higher stomatal density was observed on the abaxial surface [4]. Interestingly, the stomatal density is also regulated by various genes and have been reported in other crops too [44, 45, 46]. Being sciophytic this plant shows high nitrogen content/leaf area, high leaf mass/leaf area unit, thicker leaves having higher respiration rate and chloroplasts under full irradiance compared to net shade plants [47].

**Fig. 1:**
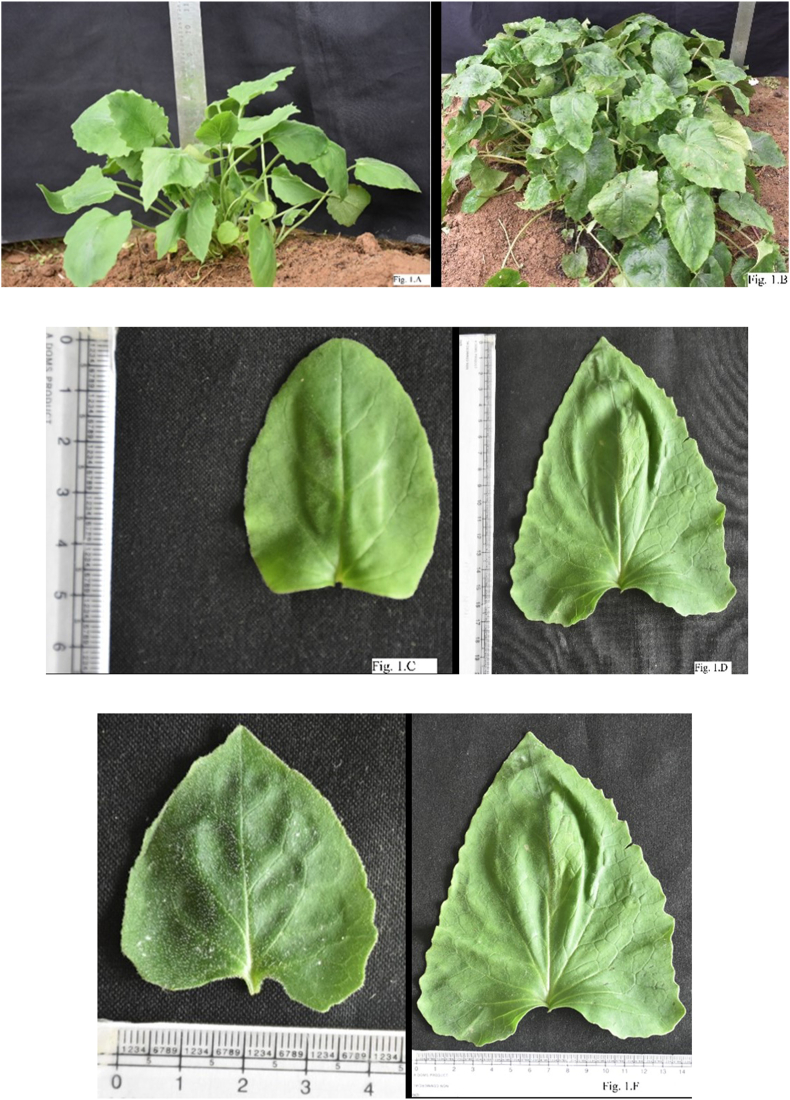
Variations in *Valeriana jatamansi* for leaf number and size in different accessions of same age 1.A) Plant with lesser leaves; 1.B) Plant with more leaves; 1.C) Shorter leaf length; 1.D) Longer leaf length; 1.E) Shorter leaf width; 1.F) Longer leaf width.

*Valeriana jatamansi* have leaves with five different types of lamina viz; oval, oval and reniform, deltoid, oval and oval-deltoid. Four different types of leaf apex viz; acute, obtuse, acuminate and acute-obtuse. Lamina base is mainly of three types; cordate, truncate with straight base and sagitate with overlapping base. Leaf margins are found to be of four types viz; entire, serrate, crenate-serrate and entire sinuate. Thakur et al [48] studied the variation in leaf characters and observed that all leaf characters are stable except leaf base and margin. The leaf margin and leaf base could be a highly plastic character which changed according to the growth and development of a plant. Chakraborty et al [42] categorized KVJ-1, KVJ-2 and KVJ-3 as entire margin, sinuate margin and wavy margin respectively. The number of leaves/plant ranged from 23-41 for the female population and 16–32 for the hermaphrodite population. The leaf length and leaf breadth varied from 5.5-9.5 cm and 4.5–7.8 cm respectively for the female population while 6.5–8.2 cm and 5.3–7.4 cm in the case of the hermaphrodite population. In 29 breeding lines of *V. jatamansi* developed through the progeny selection approach at CSIR-IHBT, Palampur (Himachal Pradesh, India) the number of leaves varied from 70-448 after two years of growth, while variations in leaf length and leaf width ranged from 4.8-16.1 cm was 4.2–14 cm (Fig. 1A–F).

The rhizome length varies from 5-20 cm having nodes and internodes [4]. Vascular bundles in rhizomes are in the circular ring having a broad parenchymatous pith with a collateral type of vascular bundle in the rhizome. However, in North Eastern Himalayas, the rhizome length/plant varied from 3.2-5.1 cm and 3.5–6.2 cm in case of female and hermaphrodite populations, respectively, while variation in the case of rhizome width/plant was 0.2–1.3 cm and 0.3–1.3 cm for female and hermaphrodite were observed, by Chakraborty ***et al.*** [42]. Karnwal ***et al.*** [49] evaluated the biomass yield of different sex forms and found that dry rootstock biomass/plant was maximum for the hermaphrodite plant (13.43) compared to the female plants. Thakur ***et al.*** [40] also compares the biomass of female and hermaphrodite plants. During this comparative study, it was found that maximum rootstock biomass (fresh 68.3 gm) and (dry 19.84 gm) weight/plant were in female plants which were statistically at par with (fresh 61.3 gm) and (dry 17.85 gm) weight of hermaphrodite plants.

**Fig. 2:**
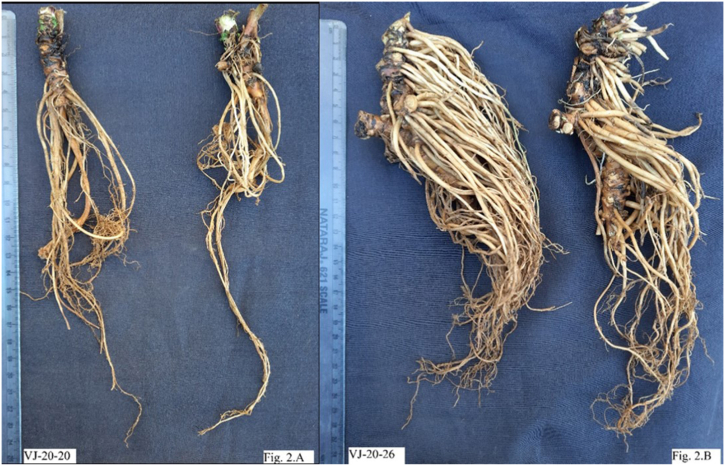
Variations in *Valeriana jatamansi* for rhizome size A) Small rhizome size; 2.B) Big rhizome size.

A wide range of variations among the number of rootstock divisions after two years of cultivation was observed at CSIR-IHBT, Palampur in the 29 selected lines of *V. jatamansi* [50]. It was found that the number of rootstock division were minimum in VJ-20-19 (6 Nos.) and maximum in VJ-20-01 (31 Nos.). The selected lines have shown vast diversity for the fresh rhizome biomass. Breeding line VJ-20-16 have yielded 480 gm of fresh underground biomass during the onset of the third year which is a significant breakthrough following VJ-20-06 (400 gm), VJ-20-18 (300 gm), VJ-20-24 (240 gm) and VJ-20-26 (190 gm). Minimum fresh underground biomass during the onset of the third year was recorded with VJ-20-20 (105 gm), VJ-20-28 (100 gm), VJ-20-29 (90 gm) and VJ-20-14 (40 gm). It was also observed that in plants of the same age, few lines were having small size of rhizomes while some lines have excellent rhizome size which may also lead to variations for valepotriate and essential oil content (as seen in Fig. 2A, B.)

### 3.2 Floral biology and pollination behaviour

**Fig. 3:**
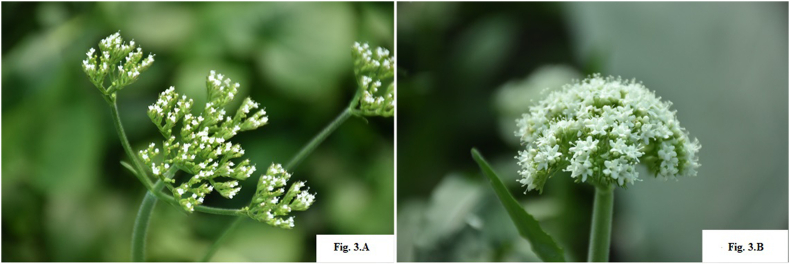
Type of flowers; 3.A Female flower, 3.B Hermaphrodite flower.

*Valeriana jatamansi* is gynodioecious herb, where female as well as hermaphrodite flowers are found on different plants. Female flower (Fig. 3A) is pistillate and has five petals which are smaller in size having trifid stigma. Hermaphrodite flower (Fig. 3B) consist both androecium and gynoecium having five petals which are larger in size with unifid stigma, inferior ovary and single ovule [[51], [52], [53], [54]]. Actinomorphic symmetry and dimorphism has been reported [52] where pink flowers developed in open sunlight and white coloured flowers developed in shade conditions, producing achene type of seeds at maturity. Entomophily is commonly observed during flowering season leading to random out-crossing and pollination of pistillate flowers enforcing cross-fertilization in natural populations. However, self-pollination is not restricted in plants with hermaphrodite flowers, and even in the absence of insect activity (physical isolation/bagging of the plant) seed-set is obtained on account of stylar movement involving a unique mechanism in which it bends in a bow shape towards the anthers or towards the corolla lobe to allow stigma to trap the pollen fallen on its surface during anthesis [51]. Seeds were successfully obtained in plants with hermaphrodite flowers when kept in isolation to check extent of self-pollination [55]. In contrast, this mechanism of stylar bending was not evident in case of unpollinated pistillate flowers. No seed-set was observed in plants with pistillate flowers when bagged or put in physical isolation, ruling out the possibility of apomixis.

The asynchronous nature in anthesis of hermaphrodite flowers allows pollen availability for longer time ensuring effective pollination with the presence of a sterile flower at every dichotomous branch of inflorescence while female set seeds due to outcrossing [56]. Female flowers are smaller to hermaphrodite flowers in size and normal chromosome segregation was seen during anaphase while observing pollen mother cell meiosis without abnormalities like laggards, bridges or micronuclei [57]. Protandry has been observed in the hermaphrodite flowers in which, stamen develop earlier than stigma to promote cross-pollination.

### 3.3 Ploidy variations

Large variations in the ploidy level have been reported in *V. jatamansi* [58]. Chromosomes may be in copies of two, four or more sets and are referred to as diploid or tetraploid based on the set of chromosomes. From the meiotic studies which were carried out using flower buds, it was found that the different populations of North-Western Himalayas have three different ploidy levels; diploid (2n = 16), tetraploid (2n = 32) and octoploid (2n = 64) in which diploid was reported as the latest addition in India. Significant variations were observed among all the different cytotypes for qualitative characters such as pollen grain size, leaf size, stomatal size, stomatal frequency, guard and subsidiary cells. Significant variations were also observed for leaf size in all cytotypes [58]. The size of the pollen grain in the octaploid cytotype was observed to be significantly larger than the diploid cytotype [58].

In the case of *V. officinalis commonly known as Valerian*, subspecies officinalis and collina were studied for cytotypes and both subspecies were subdivided into diploid and tetraploid cytotypes having 2n = 14, 28 [59]. A hexaploid ploidy level for a Pyrenean population in Spain was reported to make a new addition worldwide [60, 61]. Another species of the same genus, *V.* sambucifolia is reported to have 2n = 56. Rare disturbances during the first meiotic division were observed except for the delayed separation of some pairs [62]. When the reciprocal crosses of di, tetra and octoploid *V. officinalis* were carried out surprising results were found like a triploid progeny when an octoploid mother was crossed with a diploid father where a pentaploid was expected and tetraploid progeny of tetra and diploid parents where a triploid was expected. This may be due to improper emasculation or distorting effect during meiosis [63]. However, there is a dearth of information regarding the frequency of such polyploids in natural populations. *V. jatamansi* appears to be in an evolutionary transitional phase and the build-up of polyploid plants in the population will be possibly limited owing to modified meiotic behaviour and the number of diploids will mostly outnumber the polyploids. However, in the case of *Valeriana officinalis*, as an exception, the majority of valerian under cultivation are tetraploids.

In terms of genetics, inheritance of traits and identification of the homozygous plant in segregating population of tetraploids is more complex as compared to diploids. In diploid segregating populations, the frequency of alleles will be for three combinations (AA, Aa and aa) while for tetraploids it will be five allele combinations, viz., nulliplex (aaaa), simplex (Aaaa), duplex (AAaa), triplex (AAAa) and quadruplex (AAAA) [64, 65]. Also, to achieve homozygosity (up to 97%) in tetraploids, 21 generations will be required to be advanced through selfing (starting from duplex genotype), whereas the same would be achieved in the sixth inbred generation in diploids. A major limitation in tetraploids of *Valeriana officinalis* is in form of high inbreeding depression which impedes achieving homozygosity. Such a limitation can be successfully avoided in the diploid populations of *V. jatamansi* for the development of inbreds.

There is a high degree of polymorphism in internal transcribed spacer-region (ITS-region) sequences which can be used for genetic variability identification and are often used for genealogical studies. If there is a mutation that occurred in the tandem-like sequences of internal transcribed spacer-region through molecular approaches known as “concerted evolution” then this mutated sequence will replace all other tandem sequences or the mutated sequence will be eradicated [66]. In allopolyploids, the concerted evolution happens in bivalents while sequence variants remain permanent. The ITS-region of single plants of different *Valeriana officinalis* cytotypes were analysed and compared with available sequences from NCBI-GenBank [67]. In the ITS regions of tetra and octoploid plants, sequence variants were found. The results of genotyping and internal transcribed spacer-region analysis give an idea of allopolyploid origin, at least a step to tetraploid level from diploid [68, 69]. Such studies also need to be carried out to establish the origin of ploidy variations (auto/allo-polyploidy) in *V. jatamansi*.

### 3.4 Seed raised populations in cultivation vs clonal plants

*Valeriana jatamansi* is a sciophytic, hydrophilic plant with shallow roots which can be cultivated in fertile humus-rich loamy soil with good drainage. Roots are shallow and readily absorb moisture from irrigation or rainfall. It can be propagated by seeds as well as clonally through splitting the rhizome, cuttings of apical buds and through micropropagation [51, 55]. The seed weight/plant of females ranged from 0.3- 0.45 gm and for hermaphrodite plants it ranged from 0.22-0.37 gm Chakraborty et al [42]. The test weight of *V. jatamansi* seeds was found to be 0.329 gm. Seeds start germinating after 7–10 days and should be transplanted when they attain the 5–7 leaf stage after 3 months [4]. Seed-raised populations show a wide range of variations for phenotypic traits such as rhizome biomass accumulation (40–480 gm), the content of valepotriates (2.05–3.41%) and also EO content (0.1–1.23%).

**Fig. 4:**
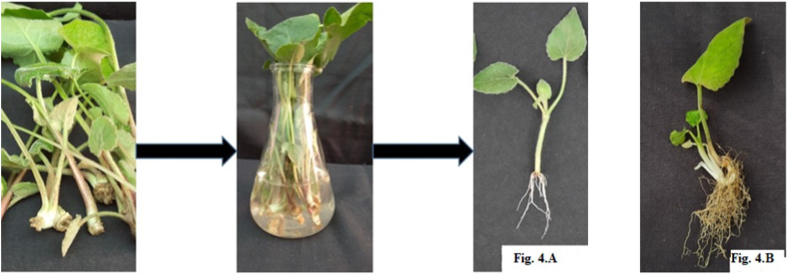
Vegetative propagation in *Valeriana jatamansi* 3. A) using apical buds; 3.B) propagation using whole leaf along with petiole.

Clonal propagation of plants is done through the splitting of the rhizome into different parts. Genotypic variations were observed for the number of rhizomes after splitting (6–31) as well as size (5–16 cm) and weight of rhizomes (40–480 gm). However, low phenotypic variations were observed within the clonal lines. Various micropropagation techniques have also been developed for clonal propagation of *V. jatamansi*. Kaur *et al.* [70] established plants by culturing shoot buds on BA and IAA or NAA nutrient-supplemented medium and established plants within 90 days in the field. Das *et al.* [71] developed callus-mediated organogenesis for *V. jatamansi* production by IBA, NAA, 2,4-D fortified Murashige and Skoog (MS) medium. Callus was induced from leaves on MS + 1.0–7.5 μM 2,4-D + 5.0 μM NAA and adventitious roots were formed by using 5.0 μM IBA, 5.0 μM NAA or 1.0–5.0 μM 2,4-D [72]. Treating nodal explants in the combination of 1.5 μM BAP, 0.5 μM NAA and 0.1 μM GA3 resulted in a hundred per cent rooting with higher root length and root number [73]. However, *in vitro* propagation is costly as compared to vegetative propagation methods. Recently, a method of vegetative propagation has been developed by treating apical shoots with a 50 mg/L concentration of NAA hormone for 30 min [55]. By using this method, it was observed that uniform crop stands were obtained and that the EO quality remained the same in the propagated plants which can fetch higher returns. Plants could also be propagated using the whole leaf along with the petiole (Fig. 4A, B).

### 3.5 Phytochemical variations

Valepotriatets from roots and rhizomes of *V. jatamansi* are of targeted interest [[74], [75], [76]], Iridoids [27, 77] flavonoids 5.75 mg/g and 5.08 mg/g for planted and wild populations, flavone glycosides viz; 6-methylapigenin, hesperidin and linarin-isovalerianate [3, 15, 78, 79, 80], thirteen different lignans [81], tannins [3], sesquiterpenoids [82, 83], bakkenollide type sesquiterpenoids [81, 84], phenolics like hydroxybenzoic acid (390.58 mg/100g), caffeic acid (158.56 mg/100 g) in the planted individual while catechin (229.59 mg/100 g), chlorogenic acid (5.52 mg/100 g), coumeric acid (2.89 mg/100 g) and gallic acid (8.70 mg/100g) in the wild plants from Kosi-Katarmal region of Uttarakhand [5] and EO [[85], [86], [87], [88]]. EOs are synthesized in the plant kingdom and are essential for physiology for its metabolism and preset developmental differentiation program of synthesizing tissues [89, 90]. EO of *V. jatamansi* has antioxidant and insecticidal properties while moderate neuroprotective effects and inhibitory activity on acetylcholinesterase were shown by iridoids and sesquiterpenoids [[91], [92], [93]].

#### 3.5.1 Iridoids

**Fig. 5:**
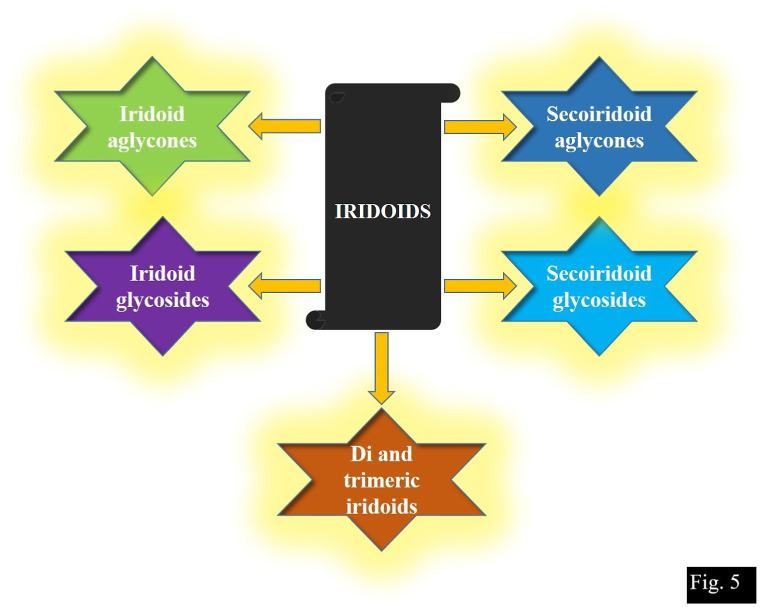
Classification of iridoids.

Iridoids [94], found in many plant families are cyclopentane-c-pyran monoterpenoids are often present in form of glycosides [95]. The classification of iridoids is given in Fig. 5. Various insects belonging to orders like Hemiptera, Hymenoptera, Lepidoptera and Coleoptera feed on iridoid-containing plants, sequester and use them to increase their reproductive behaviour or to use as a defence against predators. These are widely used as medicine for skin disorders, hypotensive, sedatives, diabetes and inflammatory diseases [96, 97]. Liu et al. [29] extracted iridoids using dried roots and rhizomes in 95% EtOH which is further vacuum concentrated resulting in the crude extract. Isopatrinioside a new secoiridoid glycoside isolated by Tan et al. [26] was monocyclic iridoid and due to cleavage of the pyran ring, it was opened between C-1 and C-2 which were found to be effective for neuronal cell death induced by CoCl2. Jatamanvaltrates R, S and Jatamanin Q were isolated in which Jatamanvaltrate S was the iridoid with fatty acid esters [93].

#### 3.5.2 Valepotriates

Valepotriates (*Valeriana*-epoxy-triesters), found in the family Valerianaceae, chemically belong to group iridoid glycosides and are important secondary metabolites which exhibit significant biological activities due to the presence of acyloxy group linkages. Roots, rhizomes or whole plants could be used for the extraction of valepotriates [81]. Being unstable compounds they are thermolabile and decompose under acidic or alkaline conditions in water and alcohol [98]. Valepotriates have therapeutic attributes [99]. Considerable progress has been made in the earlier years of the research of valepotriates and about 145 compounds have been discovered [9]. The composition of valepotriates significantly varies among parts of the plant as well as its habitat [100]. Furthermore, valepotriate content was reported to be higher in rhizomes growing under different conditions [85].

Valepotriates in leaves of *V. jatamansi* are differentiated into four groups based on their chemical structure *viz;* the diene type, the monoene type, the valtrate-hydrine type and the desoxy monoene type [74]. Various valepotriates have been categorized in Table 1. The valepotriate content varies from 2.0 to 5.6% which also differs during the vegetative and reproductive phases of the plant. During the reproductive and seed setting phase, valepotriate content varied from 2.4-3.6%. Valepotriate contents were higher during October (4.7%), November (4.9%) and January (5.4%), which indicated that November and January month is appropriate for harvesting *V. jatamansi* for valepotriate [80]. Thakur *et al.* [28] reported that valtrate is a major valepotriate. Total valepotriate ranged from 1.019-1.852% in rhizomes, 1.193–1.829% in roots and 1.094–1.801% in rootstock. Under in vitro conditions, the yield of acevaltrate and didrovaltrate can be increased using 1 mg/l of 2,4-D while 1 mg/l NAA increased the valtrate production in the callus-mediated shoot regeneration system [71]. Valepotriate content was studied by Becker & Chavadeoi [75] in normal versus cultures of cell suspension treated with colchicine and observed up to a sixty-six-fold increase in the concentration of valepotriates in colchicine-treated cultures of *V. jatamansi*.

**Table 1:** Classification for valepotriates of *Valeriana jatamansi*.

Diene	Monoene	Valtrate-hydrine	Desoxy monoene
Valtrate	Didrovaltrate	*Valtrate hydrine* B1	8,11-Deoxididrovaltrate
Isovaltrate	Isodidrovaltrate	*Valtrate hydrine* B2	8,11- Desoxihomodidrovaltrate
Acevaltrate	IVHD valtrate	*Valtrate hydrine* B3	
Diavaltrate	Homodidrovaltrate	*Valtrate hydrine* B4	
Homoacevaltrate	AHD valtrate	*Valtrate hydrine* B5a	
1-homovaltrate		*Valtrate hydrine* B5b	
7-homovaltrate		*Valtrate hydrine* B6A	
11-Aceavltrate		*Valtrate hydrine* B6b	
Hydroxyvaltrate		*Valtrate hydrine* B7	
Isohomoacevaltrate		*Valtrate hydrine* B8	
Deacetylisovaltrate			

The diene valepotriates are stable at 20 °C when kept in anhydrous methanol. Storing valepotriates at room temperature with little water dissolved in methanol or ethanol decomposes 90% in a few weeks, giving yellow-coloured baldrinal as a decomposition product [98, 101]. Two new valepotriates; jatamanvaltrate T and jatamanvaltrate U were discovered and jatamanvalterate T was found to be an allosteric modulator and could play a crucial role in attenuating nociception [76].

Singh *et al.* [34] evaluated twelve accessions of *V. jatamansi* for variations in valepotriates and concluded that the valepotriate content from different locations of Himachal Pradesh, India varied from 1.2% to 1.9%. Sharma and Mondal [102] studied the field performance of *V. jatamansi* and found that room-dried roots from higher altitudes contained 0.426% valerenic acid as compared to the lower altitude (0.186%). Gehlot *et al.* [103] studied the production of valerenic acids under the submerged cultivation of *V. jatamansi* and found that a higher accumulation of valerenic acid (1525.14 μg/g) was in plant rhizomes whereas acetoxyvalerenic acid (543.91 μg/g) and hydroxyl valerenic acid (919.57 μg/g) was higher in adventitious roots. It was also found that the maximum fresh root biomass was with half-strength Schenk & Hildebrandt medium having 2% sucrose and 4.92 μM IBA. Shukla *et al.* [30] analysed the phytochemicals of *V. jatamansi* from different locations in Jammu & Kashmir, India and found that the sample from Patnitop contain 0.38 mg/g and that from Sanasar contained 0.27 mg/g of valerenic acid. In a study conducted by Jugran *et al* [3] in Uttarakhand, India higher valerenic acid in aerial parts (0.57 ± 0.04%) was reported from Katarmal region while higher content of valerenic acid (1.80 ± 0.12%) from root portion was reported from the Joshimath region. As per the Indian Council of Agricultural Research report, it was found that valepotriate is found in the cortex of rhizome, root and rhizome pith which with maximum content of 3.56% and valtrate being the main component [104]. In a different study, wild collections of *V. jatamansi* from different locations of Shimla, Sirmaur, Chamba and Solan districts in Himachal Pradesh, India were analysed and maximum valepotriate content was found in the rhizome (3.41%), root (3.74%) and rootstock (3.57%) of plants from Dalhousie of Chamba district. The range of valepotriates varied from 2.05-3.41% in the case of rhizome, 2.18–3.74% in root and 2.11–3.57% for rootstock [105]. It was also observed that the content of valepotriates decreased significantly with an increase in storage duration. The proportion of some important valepotriate [105] in different parts of the plant is presented in Table 2.

**Table 2:** Valepotriate content (%) of different plant parts from various locations.

	Rhizome				Root				Rootstock			
Location	Valtrate	Didrovaltrate	Acevaltrate	IVDH Valtrtae	Valtrate	Didrovaltrate	Acevaltrate	IVDH Valtrtae	Valtrate	Didrovaltrate	Acevaltrate	IVDH Valtrtae
Shogi	1.47	0.19	0.33	0.21	1.56	0.20	0.35	0.24	1.54	0.19	0.34	0.22
Kotkhai	1.43^#^	0.18	0.34	0.24	1.57	0.23	0.35	0.28	1.46	0.21	0.34	0.26
Habban	1.96	0.19	0.35	0.25	2.05	0.20	0.39	0.27	2.01	0.19	0.37	0.26
Dalhousie	2.39*	0.28*	0.39*	0.35*	2.60*	0.33*	0.43*	0.38*	2.48*	0.31*	0.41*	0.36*
Kalatop	2.11	0.23	0.32	0.26	2.24	0.29	0.40	0.33	2.19	0.27	0.36	0.29
Kufri	1.50	0.17	0.29	0.19	1.67	0.15^#^	0.29^#^	0.21^#^	1.60^#^	0.16^#^	0.29	0.20^#^
Mashobra	1.46	0.15#	0.26^#^	0.18^#^	1.52	0.18	0.30	0.21^#^	1.48	0.17	0.25^#^	0.21
Chail	1.60	0.16	0.28	0.23	1.71	0.19	0.34	0.27	1.66	0.17	0.31	0.25
Chhajpur	1.76	0.20	0.33	0.28	1.96	0.25	0.36	0.32	1.85	0.23	0.34	0.30
Khadapather	1.46	0.15#	0.29	0.21	1.47^#^	0.17	0.30	0.24	1.47	0.16^#^	0.29	0.23
Range	1.43–2.39	0.15–0.28	0.26–0.39	0.18–0.35	1.47–2.60	0.15–0.33	0.29–0.43	0.21–0.38	1.60–2.48	0.16–0.31	0.25–0.41	0.20–0.36

#signifies lower content, * Signifies higher content; Gupta (2001) [105].

#### 3.5.3 Variations in EO composition

Studies on EO from rhizome and roots of *V. jatamansi* led to the identification of major compounds which are maaliol, patchouli alcohol, calarene/ß-gurjunene, seychellene, α-santalene [9, 24]. Other compounds present were α-patchoulene, viridiflorol, α-guaiene, kessane, α-bulnesene/δ-guaiene, spathulenol, bornyl acetate, 7-epi-α-selinene, ß-patchoulene [24, 106]. In the leaf oil, the major constituents were 3‐methyl valeric acid and maaliol however, the maximum content in root oil was found to be maaliol and β‐gurjunene [23]. Unidentified sesquiterpene hydrocarbon was detected by Bos *et al.* [107] with identified molecules like ar‐curcumene, xanthorrhizol and α‐santalene in the root and rhizome oil of *V. jatamansi* of European origin while the main component in case of Nepalese and commercially available root material is patchouli alcohol. From the wild-grown population's variation in quantity and quality of EO was noticed, roots giving higher yield (0.35–0.43%) than rhizomes (0.05–0.08%); α-bulnesene, α-guaiene, bornyl acetate, 7-*epi*-α-selinene, γ-patchoulene and β-elemene being a major proportion in root oil while patchouli alcohol, maaliol, isovaleric acid and viridiflorol quantity was higher in rhizome oil [86]. The studies conducted by Singh *et al.* [85] revealed the influence of season on the quantity of EO and found May to be the promising month for EO yield from rhizomes. Phenolic concentration, antioxidant activity and flavonoids were found to be higher during the pre-flowering stage. An inverted proportion was also observed for the concentration of mono and sesquiterpenes with oxygenated sesquiterpenes among phenological stages with different altitude gradients [108].

He *et al.* [2] separated volatile profiles of collected samples from the wild and common garden which demonstrated that the environmental factors strongly affected the chemotypes. It was found that the six volatile compounds differentiated wild and common garden samples. Singh et al [87] reported an essential oil range of 0.6%–1.66% with a negative correlation between patchouli alcohol and viridiflorol. A positive correlation between patchouli alcohol was found with alpha-guaiene, seychellene and azulene. Population like Chamba-II was excellent in viridiflorol (48.8%) and Kandi-I (65.04%) was good for patchouli alcohol.

As revealed in the study by Gautam *et al.* [55] the essential oil components of two lines; clones of CSIR-IHBT-VJ-05 with Himbala and have shown chemotypic distinction due to their diverse characteristics and geographic origin. With the detection of nine components among the two populations, it was found that the major compound, patchouli alcohol was 62.38% in the clone and 33.35% from the Himbala's seed-raised plants. Interestingly, fifteen compounds were identified in the cloned and mother plant of CSIR-IHBT-VJ-05 that have shown non-significant results viz; patchouli alcohol (62.38 and 62.89%), seychellene (4.74 and 4.82%) and alpha-guaiene (3.01 and 3.03%) being the main. This suggests that the quality and homogeneity of essentials could be maintained by clonal multiplication. Patchouli alcohol is one main component of *V. jatamansi* EO forming the largest fraction (about 62%). Studies based on EO exhibited wide variations reaching up to 65.04% of patchouli alcohol in the oil samples analysed [24, 55, 109]. However, certain accessions from Bageshwar and Dehradun (Uttarakhand, India) are reported to be rich in maaliol (53.8%). These accessions have low levels of patchouli alcohol in the rhizomes [24]. Such variations in the quality are important to achieve EOs of different grades rich in particular chemical constituents and selection for these traits will be effective for the development of superior varieties from a quality point of view. Variations in open pollinated variety (Himbala) for major compounds in EO are presented in Table 3 [55].

**Table 3:** Variations for major compounds in EO; Gautam *et al.* (2021) [55].

Compound	Range (%)
Patchouli alcohol	30.67–63.39
Santalene	0.58–7.02
Guaiene	2.74–3.06
Trans-a-bergamotene	3.89–4.07
Azulene	5.04–6.86
Cis-caryophyllene	15.4–16.2
Seychellene	4.72–5.05
Curcumene	6.49–7.29

In the chemical diversity analysis by Thakur et al [110] it was found that patchouli alcohol is the main compound and ranged from 19-63.1% irrespective of the population and distillation day. Patchouli alcohol content was found to be maximum during the first day and declined sharply as the number of days increased. It was very interesting to know that the amount of sychellene increased with the days of extraction irrespective of the population. Moreover, delta-guaiene concentration increased on the second and third days of distillation. Four promising lines for patchouli alcohol content were identified viz; VJ-20-11 (65.1%), VJ-20-04 (65.01%), VJ-20-12 (64.68%) and VJ-20-02 (62.92%) in studies conducted by Gautam and Singh [50].

## 4 Breeding objectives for population improvement

*Valeriana jatamansi* is in high demand, both in the aromatic and pharmaceutical industry. To promote its organized cultivation, breeders should develop varieties with increased EO and valepotriates content. Since, EO content and valepotriates are found in roots and rhizome of *V. jatamansi*, high biomass of roots and rhizome constitutes an important breeding objective. The second important issue that needs to be addressed is uniformity of the produce. In both the cases of collections from the wild and open pollinated varieties, heterogeneous raw produce is a major limitation for aromatic and pharmaceutical industry, which is further compounded by the perennial life-cycle of the plant as it is mostly harvested after two years of transplantation [85]. Varieties with uniform produce and early maturity are required to meet the specific needs of the industries. For instance, major components like patchouli alcohol and maaliol are present in rhizome rather than roots [86] making rhizome the desirable material for aromatic industry, while dense root system (Fig.r 6(b)) tends to increase the post-harvest cost in terms of washings required to remove soil and may in turn also affect the oil quality. Overall, the breeding objectives are high root and rhizome biomass, high content of secondary metabolites and EO, early maturity and uniformity of the produce.

**Fig. 6:**
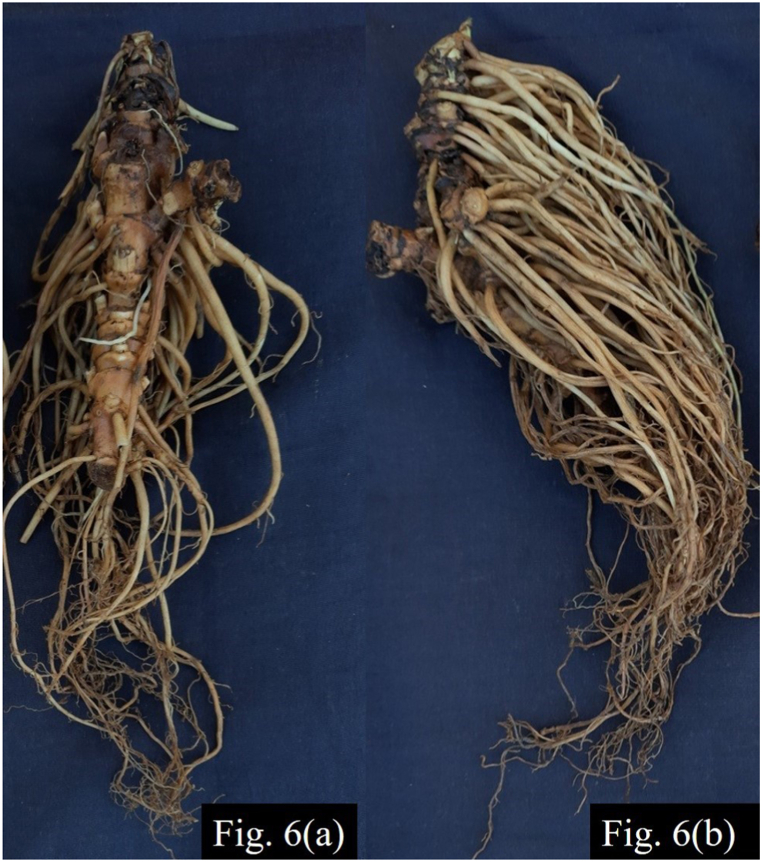
(a). Sparse and (b) dense roots in *Valeriana jatamansi*.

### 4.1 Breeding techniques

It is evident that *V. jatamansi* is a cross pollinated crop, so pollination control becomes the first and main step for achieving the breeding objectives. Self-pollination of plants bearing hermaphrodite flowers is possible through isolation/bagging at the onset of flowering and seeds have been successfully obtained through this method [55]. Cross-pollination of plants bearing pistillate flowers is also feasible using pollens obtained from selected hermaphrodite plants, although, isolation also needs to be maintained in this case to prevent contamination from other pollen sources. In case of crosses involving plants with hermaphrodite flowers, small size of flowers makes it difficult to emasculate, thus making crosses a time consuming process and rate of success and seed production after emasculation is reported to be low [, so crosses utilizing emasculation technique can be used in scientific investigations [111]. Hybridization has been successfully used to combine desirable characteristics in breeding *Valeriana officinalis* [68]. The temperature sensitivity of male and female parts of *Valeriana officinalis* has minimal difference which eliminates the use of hot water treatment for emasculation [112]. The genetic identity of phenotypically and chemically characterized lines needs to be maintained and that can be achieved clonally [55].

### 4.2 Collection and conservation of genetic resources

A crop improvement program in a species is based on the diverse and variable genetic resources. Exploration is an integral activity of breeding programs followed by collecting genetic resources and unique germplasm lines can directly be developed as a variety/cultivar of that species. Wide range of phenotypic and chemical diversity is observed in germplasm collections of *V. jatamansi* [40, 42, 55]. Some of the elite germplasm lines have been listed by different researchers and also registered with PGRC, National Bureau of Plant Genetic Resources (NBPGR), ICAR, New Delhi (Table 4).

**Table 4:** Various accessions and registered germplasm of *Valeriana jatamansi*.

Accession number	Collection site	State	Unique trait	Research group
IC0630604, INGR20096	Salooni, District Chamba	Himachal Pradesh	High root biomass	Singh et al. (2022) [113]
IC0630605, INGR20057	Banjar, District Kullu	Himachal Pradesh	High Patchouli alcohol in EO (62.38%)	Kumar et al. (2022) [114]
IC 582516	Ginoti (Uttarkashi)	Uttarakhand	1.67 % EO	Raina and Negi (2015) [24]
IC 573221	Kanda (Bageshwar)	Uttarakhand	1.71 % EO	Raina and Negi (2015) [24]
IC 573222	Kanda (Bageshwar)	Uttarakhand	2.00 % EO	Raina and Negi (2015) [24]
IC 573210	Niglat (Nainital)	Uttarakhand	1.06 % EO	Raina and Negi (2015) [24]
IC 573212	Dunagiri (Almora)	Uttarakhand	1.20 % EO	Raina and Negi (2015) [24]
IC 589096	Mussorrie (Dehradun)	Uttarakhand	0.89 % EO	Raina and Negi (2015) [24]
IC 574522	Shillong (Shillong)	Meghalaya	0.64 % EO	Raina and Negi (2015) [24]

*Ex situ* conservation is an important approach for conserving genetic resources in form of germplasm accessions. *Ex situ* conservation involves field gene bank of living plants of vegetatively propagated crops. Mukherjee *et al.* [115] studied the mode of *ex situ* conservation in *V. jatamansi* at high altitudes at four different locations to determine the survival rate and found that *V. jatamansi* has shown higher survival rate at higher altitude range 2000–3000 mean sea levels as compared to lower altitudes. As a backup, *in vitro* conservation of germplasm is also important to protect the unique genetic resources from risk of extinction in case of inclement weather conditions. Normah *et al.* [116] found that *in vitro* culture has offered disease-free multiplication of clonally propagated *V. jatamansi*. Sharma *et al.* [117] studied the mode of cryopreservation in *V. jatamansi* in which shoot tips were conserved using a mixture of two cryoprotectants and observed that cryopreservation can be used for long term conservation of *V. jatamansi* and shoot tips were treated with Plant Vitrification Solution-2 at 0 °C for 110 min which showed greater shoot recovery as compared to others. In this way, germplasm of *V. jatamansi* has been conserved by encapsulation of somatic embryo, shoot tips and axillary buds.

### 4.3 Selection

Selection is an important tool which gives direction to the breeding program and effective selection depends on variability among the parental lines, selection intensity and heritability of the traits of interest. Most of the information pertaining to variability in *V. jatamansi* is available for the natural populations occurring in the wild. Heterogeneity of such populations on account of random out-crossing, perennial growth habit, age of plants in the same population and seasonal variations in the environment, makes it difficult to screen the plants at an early stage. Evaluation of the biomass yield in *V. jatamansi* plants was done by Karnwal et al. [49] to identify maximum dry rootstock biomass/plant and similarly, Thakur et al. [40] also evaluated the biomass of rootstock on fresh (68.3 gm) and dry (19.84 gm) weight/plant basis for identifying potential selections. Based on progeny selection approach in *V. jatamansi* germplasm collections at CSIR-IHBT, Palampur (Himachal Pradesh, India), breeding line VJ-20-16 was identified having 480 gm of fresh underground biomass [50]. Response to selections based on progeny test hold potential in improving the rootstock biomass of *V. jatamansi* as it utilizes the heritable component of the phenotypic traits.

Environmental variations affect the production of secondary metabolites and therefore, selection for quality traits may not be effective when the data is available for a particular season or location [55]. Similar observations are available for *Valeriana officinalis* where, the contents are influenced by environmental conditions, plant development stage, processing strategies and genetic constitution as well [[118], [119], [120], [121]]. A correlation could be established between the EO in *Valeriana officinalis* and the plant stage in which it was obtained. It was found that EO of *Valeriana officinalis* increased in the vegetative phase and decreased with the formation of generative parts [[118], [119], [120], [121]]. The amount of valerenic acid was highest in or just before the flowering period [119, 121], while Bernath [120] discussed that the growth factors (light, temperature, water requirement, soil conditions, nutrition) also influenced secondary compound production.

### 4.4 Population improvement

On account of unique floral dimorphism in *V. jatamansi*, there is a tendency towards natural cross-pollination (xenogamy). Accordingly, breeding methods for population improvement are applicable to Indian valerian. The simplest method for population improvement is mass-selection in which phenotypically superior plants are selected in the population, followed by seed production by random open pollination among the selected plants. This method does not involve progeny testing and is comparable to open pollinated variety. When selection of phenotypically superior plants is based on performance of their progeny lines (such as ear-to-row method) it is termed as mass selection with progeny test which provides higher level of selection efficiency to the breeding program.

### 4.5 Recurrent selection

With a focus to utilize heterosis in cross-pollinated crops, recurrent selection schemes have been devised [122] to ensure the isolation of superior inbreds from populations subjected to recurrent selection. Basically, these schemes are extension of progeny selection approach depending on the manner of obtaining progenies for evaluation and making crosses in all possible combinations in comparison to random mating in case of open pollination. In *V. jatamansi* recurrent selection may help in increasing the underground mass as well as EO and valepotriate content.

### 4.6 Simple recurrent selection

It is a type of recurrent selection in which a number of plants with desirable phenotype are selected and self-pollinated followed by raising of separate progeny rows from selfed seeds of selected plants. The progenies are inter-crossed in all possible combinations and equal amount of seeds from each cross is composited to produce the next generation. Population is subjected to more than one recurrent selection cycle to increase the frequency of desirable traits in the population.

### 4.7 Recurrent selection for general combining ability

In this scheme, progenies of selected plants are obtained by crossing selected plants to a tester parent with broad genetic base and also self-pollinated. This scheme is based on early testing method suggested by Jenkins [123] which involves testing of inbreds for combining ability in early stages of inbreeding. Final selection of superior plants is based on evaluation of test-cross progeny lines in replicated yield trial for general combining ability of lines. The self-progenies of superior selected plants are crossed in all possible combinations and equal amount of seed from each cross is composited to obtain the subsequent generation. This selection cycle needs repetition to improve the yielding ability of the population in terms of increasing the underground mass as well as EO and valepotriate content in *V. jatamansi* Indian valerian.

### 4.8 Recurrent selection for specific combining ability

The purpose of this method is to isolate potential lines from a population that combine well with a given inbred. It was first proposed by Hull [124] with focus on specific combining ability. In this case progeny of selected plants are obtained by crossing selected plants to a known inbred with outstanding performance and the selected plants are also self-pollinated. Superior plants are selected based on performance of test-cross progeny lines in replicated yield trial. The self-progenies of superior selections are crossed in all possible combinations and equal amount of seed from each cross is composited to obtain the subsequent generation. This selection cycle is further repeated to isolate superior lines which combine well with an outstanding inbred in specific combinations for exploitation of heterosis.

### 4.9 Reciprocal recurrent selection

This method of improvement is applicable when improvement is sought in two different populations in their ability to combine with each other [125]. In this method, selected plants of one population are crossed with a random sample of plants of the other population and vice-versa. The selected plants of each population are also selfed. Superior plants are identified in both populations depending on the performance of their progenies in separate replicated yield trials of the respective populations. Selfed seeds of selected plants are raised in separate crossing blocks of the respective populations and pollinated in all possible combinations only within the blocks. In this method the strategy is to select both for GCA (involving broad genetic base testers in form of populations) and SCA (selection of plants in a population is based on their ability to combine well with the plants of the other population and ultimately the two populations would be crossed with each other for development of a new variety). Populations developed through reciprocal recurrent selection may be used for isolation of inbreds and development of synthetic variety.

### 4.10 Isolation of inbreds

Inbreds isolated from a particular population when crossed with inbreds from other population leads to expression of maximum heterosis in the hybrids. Specific combining ability of inbreds represents the non-additive component of gene action which is maximized when inbreds obtained from diverse populations are crossed. Repeated selfing is prerequisite for development of inbred lines. In *V. jatamansi*, self-progenies obtained from a hermaphrodite plant are observed to segregate for pistillate and hermaphrodite flowers. Repeated selfing of hermaphrodite plants from elite populations in each generation may lead to development of inbreds, although this possibility is yet to be investigated regarding response of the plant species in terms of inbreeding depression. Alternatively, a parallelism may be drawn among plants with pistillate flowers and male sterile lines, while hermaphrodite siblings of pistillate plants (obtained from same self progenies) will serve as maintainer lines. In fact, repeated sib-mating will be required for the development of such inbreds in *V. jatamansi* while retaining a level of heterozygosity to avoid possible inbreeding depression. These inbreds will be plants with pistillate flowers and function as male sterile lines as is the case in hybrid varieties. Using this method, inbreds can be developed from diverse populations through generation advancement in isolation and inbreds so obtained may be maintained and multiplied through clonal propagation.

An investigation was carried out on four inbred lines (I3) that were crossed in *Valeriana officinalis* and it was found that values for negative and positive mid-parent heterosis (MPH), were different between the inbreds and characteristics [126]. Observations suggest that crossings of different inbreds exhibit higher MPH as compared to crossings of same inbreds. The main aim of inbreeding is to decline allele variation and create greater homozygosity and homozygous loci in the plant material.

In *Valeriana officinalis*, major cultivars are tetraploid. According to Stoskopf *et al.* [65] identifying plants that are homozygous and determining the dominant alleles frequency is very difficult in *Valeriana officinalis* and homozygosity in tetraploids is expected to be achieved after 21 generations of selfing. However, inbreeding depression occurs in *Valeriana officinalis* in the first inbred generation itself and further increases with each generation. The vitality indicating characteristics such as seed yield, plant height, time for full ground coverage are affected by inbreeding and it had been illustrated that further inbreeding after third generation are difficult to execute and lead to the production of inviable plant material [69]. Response of *Valeriana jatamansi* to inbreeding needs to be studied in detail to isolate inbred lines.

### 4.11 Development of synthetic and composite varieties

There is a need to develop *V. jatamansi* variety with better root mass having increased valepotriate and EO content which could be produced using low cost seed raised plants. A synthetic variety can be developed by inter-crossing inbred lines derived through recurrent selection for general combining ability (utilizing additive and additive x additive components of heterosis) and also reciprocal recurrent selection (utilizing dominance, dominance x dominance and dominance x additive components of heterosis) schemes. More than one inbred lines are tested for combining ability for producing synthetic variety and seed production of synthetic variety is obtained by random crossing among the inbred lines raised in a crossing block.

In case of composite varieties, seeds from several lines of similar phenotype are mixed and allowed to open pollinate. Unlike synthetic varieties composites are developed without testing them for their combining ability but composite varieties show significantly more heterosis than open pollinated varieties. The main objective of developing synthetic and composite varieties is to utilize the available heterosis on account of high degree of natural outcrossing and lack of pollination control system. Synthetic varieties have higher yield than open pollinated variety but lower yield than single and double cross hybrids.

### 4.12 Development of hybrid varieties

A single-cross hybrid variety is developed by inter-crossing two inbred lines derived through recurrent selection for specific combining ability (utilizing dominance and dominance x dominance components of heterosis) as well as reciprocal recurrent selection (utilizing dominance, dominance x dominance and dominance x additive components of heterosis) methods. In a single cross, inbreds (I) are crossed in the order IA x IB (where A and B represent origin of inbreds from diverse populations). Alternatively, double cross inbreds may also be developed involving four inbreds in the order (IA1 x IA2) X (IB1 x IB2) to maximize yields (utilizing additive, dominance and interaction components of heterosis).

### 4.13 Doubled-haploidy breeding

Utilizing biotechnological approaches such as anther culture or chromosome elimination technique, haploid plants could be generated which can be used for production of doubled haploids through colchicine treatment. Doubled haploid lines are genetically 100% homozygous (inbreds) and can be readily utilized for development of hybrid varieties when they have a diverse origin. A protocol for doubled-haploidy breeding in a plant species shortens the breeding cycle to a single generation for developing homozygous plants. Certain attempts were made [[127], [128], [129]] for developing haploid *Valeriana officinalis* plant through anther culture, but were unsuccessful. Reciprocal crosses of di, tetra and octoploid *Valeriana officinalis* resulted in triploid progeny in a cross involving octoploid (♀) x diploid (♂). This may be due to distorting effect during meiosis [63] leading to chromosome elimination. Investigations are yet to be made in *V. jatamansi* on response to anther culture and also hybridizations involving different ploidy levels to possibly yield haploids which may be utilized for production of doubled haploids through colchicine treatment to achieve chromosome doubling.

## 5 Conclusion and future prospect

*Valeriana jatamansi* is a high value medicinal plant species. The essential oil and valepotriates obtained from the plant have high demand in pharmaceutical and aromatic industry. Besides its major applications in pharmaceuticals, the essential oil has been reported with many biological activities (cytotoxic, antispasmodic, anticonvulsant, nematicidal, anti-inflammatory etc.). Indeed, the worldwide demand of high quality planting material of *V. jatamansi* is expected to increase in the near future. Therefore, to meet the growing demand, both quality and quantity plantation material of *V. jatamansi* is urgently required. To fulfil this goal, *V. jatamansi* varieties with desired traits like higher root biomass, high oil content, uniform produce and early maturity are required to meet the specific demand of the industries. This review presents information about phenotypic and phytochemical variations in *V. jatamansi* germplasm which were successfully utilized for initial improvement of yield and quality through progeny and clonal selection approaches. Further improvements are envisaged through various breeding schemes aimed at population improvement and utilization of both the additive and dominance components of variation in the reconstituted populations developed through breeding.

There are wide range of variations for the desired phenotypic traits of *V. jatamansi* in the form of underground biomass, essential oil content and maturity of the crop among different clonal lines, indicating significant genotypic variability for the traits. These variations can be utilized for making effective selections for the desired traits. Also, there is a need to maintain the genetic identity of phenotypically and chemically characterized lines clonally. High response of the species to clonal propagation is an added advantage and can be applied for maintaining the genetic purity of unique germplasm and conservation of diverse genetic resources in form of a field gene bank. Further, the recurrent selection strategies can be employed for development of novel varieties having higher root and rhizome biomass. Response to selections based on progeny test hold potential in improving the rootstock biomass of *V. jatamansi* as it utilizes the heritable component of the phenotypic traits.

The cross-pollinated breeding behaviour of the species owing to floral dimorphism and development of selfed progenies of hermaphrodite plants obtained through controlled self-pollination presents a unique opportunity for applying the afore stated breeding methods for population improvement. The repeated selfing of hermaphrodite plants will lead to the formation of inbreds. These inbreds could be utilized for specific combining ability which could then be used for the development of hybrids. Investigations are yet to be made in *V. jatamansi* on response to anther culture and also hybridizations involving different ploidy levels to possibly yield haploids which may be utilized for production of doubled haploids through colchicine treatment to achieve chromosome doubling.

Significant variations have been observed for quality aspects (valepotriates and essential oil) based on season to season and different geographical location, signifying the role of environment on harvest quality. Moreover, right stage for the uprooting of plants need to be identified so as to produce best quality of essential oil. Selection procedures need to be devised in alignment with climatic factors during crop growth and harvesting time to ensure uniform quality of root biomass. The study aims to maximize the selection accuracy for development of breeding populations with improved biomass and higher yield of different active constituents to meet the requirements of pharmaceutical and aroma industries.

## Author contribution statement

All authors listed have significantly contributed to the development and the writing of this article.

## Funding statement

Funded by CSIR Aroma Mission - Phase III (HCP-0007).

## Data availability statement

Data included in article/supplementary material/referenced in article.

## Declaration of competing interest

The authors declare that they have no known competing financial interests or personal relationships that could have appeared to influence the work reported in this paper.
